# Paradox of COVID-19 in pregnancy: are pregnant women more protected against or at elevated risk of severe COVID-19?

**DOI:** 10.2217/fmb-2021-0233

**Published:** 2022-05-05

**Authors:** Sheila Santa, Derek A Doku, Charles O Olwal, Charles A Brown, Emmanuel A Tagoe, Osbourne Quaye

**Affiliations:** ^1^West African Centre for Cell Biology of Infectious Pathogens (WACCBIP), University of Ghana, Accra, Ghana; ^2^Department of Biochemistry, Cell & Molecular Biology, College of Basic & Applied Sciences, University of Ghana, Accra, Ghana; ^3^Department of Medical Laboratory Sciences, University of Ghana, Accra, Ghana; ^4^West African Genetic Medicine Center, University of Ghana, Accra, Ghana

**Keywords:** COVID-19, physiological adaptations in pregnancy, pregnancy, SARS-CoV-2

## Abstract

Many underlying medical conditions have been linked to worse COVID-19 prognosis. Based on reports on SARS-CoV-1 and Middle East respiratory syndrome infections, pregnancy has been considered a predisposing factor to severe COVID-19, with pregnant women being a high-risk group for several physiological reasons. Specifically, pregnant women undergo physiological adaptations that predispose them to severe respiratory viral diseases, including SARS-CoV-2. However, a significant amount of evidence suggests that the clinical outcome of COVID-19 among pregnant women is not different from the general population. In view of this, this report discusses the physiological conditions in pregnant women that adversely affect their immunity, cardiovascular homeostasis, and their endothelial and coagulopathic functions, thereby making them more prone to severe viral infections. We also discuss how these physiological adaptations appear to paradoxically offer protection against severe COVID-19 among pregnant women.

COVID-19 is a respiratory pneumonia caused by SARS-CoV-2. The severity of COVID-19 differs from person to person and is generally influenced by old age, gender, comorbidity with chronic diseases, obesity and pregnancy [1]. Although women are less affected by SARS-CoV-2 compared with men, pregnant women are considered a high-risk group due to their increased susceptibility to viral infections [2]. Notably, during the influenza pandemics of 1918, 1957 and 2009, high numbers of deaths among reproductive-age women occurred in pregnant women [3–5]. Moreover, pregnancy is associated with anxiety leading to adverse outcomes such as hypertensive disorder [6], which is a risk factor for severe COVID-19.

Against this background, it was expected that pregnant women will be relatively worse affected by COVID-19 compared with nonpregnant women of comparative age [7]. However, to date, studies on severity of COVID-19 among pregnant women have provided conflicting outcomes; it is not clear whether pregnancy is a blessing or a curse among COVID-19 patients. In this special report, we provide a balanced discussion on the current knowledge of the effects of COVID-19 on pregnancy and discuss physiological conditions in pregnancy that are expected to worsen COVID-19 outcomes and how these conditions paradoxically tend to lessen the outcomes of COVID-19 among pregnant women.

## Pregnancy-related risk factors of COVID-19

Generally, pregnancy predisposes women to more severe disease outcomes compared with nonpregnant women. In view of this, pregnant women have been considered a high-risk group for severe COVID-19. Due to physiological and immunological adaptations during pregnancy, pregnant women were expected to be more susceptible to severe respiratory illness [8,9] compared with their nonpregnant counterparts. Indeed, some reports have shown that pregnant women with COVID-19 have an increased risk of requiring admission to an intensive care unit, invasive ventilation, extracorporeal membrane oxygenation and preterm delivery compared with COVID-19-negative pregnant women [10].

In pregnancy, factors such as increased maternal age, high body mass index and pre-existing comorbidities tend to influence COVID-19 prognosis [10]. Considering that SARS-CoV-2 infection presents serious pulmonary manifestations including pneumonia (the most prevalent nonobstetric condition in pregnancy [11]), acute respiratory distress syndrome, pervasive microemboli and coagulation perturbations [12], a higher morbidity and mortality was expected among pregnant women. In addition, pregnancy is associated with normal maternal physiological changes such as hypercoagulability, altered cell-mediated immunity [13] and alterations in pulmonary function, which cause reduced total lung capacity and an inability to clear pulmonary secretions effectively [14]. These changes are expected to predispose pregnant women to clinically severe pneumonia upon contracting COVID-19.

Pregnancy is associated with altered functioning of the immune system, which is likely to elevate the risk of developing severe COVID-19. The release of proinflammatory cytokines is inhibited by hormonal cues in pregnancy, particularly increased levels of progesterone [15]. A Th2 polarization phenomenon, which involves the suppression of dominant cell-mediated proinflammatory Th1 immunity in favor of a physiological shift to humoral Th2-dominant immunity, has the potential of increasing the susceptibility to intracellular pathogens including viruses, bacteria and parasites [16]. This likely explains why pregnant women are relatively more susceptible to viral infections compared with nonpregnant women [17].

Another risk factor to severe COVID-19 is altered functioning of the respiratory system during pregnancy. An important respiratory change that occurs in pregnancy is an increased oxygen consumption, which is characterized by an elevation of the physiologic diaphragm that prompts a restriction in lung expansion, and hormone-induced edema of upper respiratory tract mucosa [18]. This makes pregnant women more sensitive to hypoxia and generally more susceptible to respiratory pathogens. As pregnancy progresses, a series of changes result in closure of small airways and subsequent reduction of functional residual capacity (FRC) and expiratory reserve volume (ERV) [19]. The respiratory changes include an upward elevation of the diaphragm due to distension of the uterus, and alteration in thoracic and chest wall configuration and lung volume [20]. There is also lengthening and reduced thickening of the muscle fiber of the diaphragm, which increases the capability of the diaphragm to generate tension. The alterations in pulmonary volumes such as FRC and ERV decrease steadily from early pregnancy due to diaphragmatic splinting by the gravid uterus, resulting in reduced total lung capacity at term as well as poor clearance of pulmonary secretions [20].

## Is pregnancy protective against severe COVID-19?

Despite pregnant women having a higher likelihood of developing severe COVID-19 complications, accumulating evidence points to the contrary, with several studies pointing to favorable COVID-19 outcomes among pregnant women [21–26]. Based on recent findings, we highlight reports that favor a worse or better COVID-19 outcome among pregnant women. Table 1 summarizes the key findings of studies on COVID-19 in the context of pregnancies.

**Table 1.** A summary of reports on COVID-19 outcomes among pregnant women.

Title of article	Study design	Key findings	Remarks	Ref.
Pregnancy outcomes in COVID-19: a prospective cohort study in Singapore	Prospective observational study	The majority (87.5%) of SARS-CoV-2-infected pregnant women had mild disease	Better COVID-19 outcome with pregnancy	[26]
Pregnancy outcomes during the COVID-19 pandemic in Canada, March–August 2020	Review of clinical data and computed tomography examination	All women in the study achieved good recovery from COVID-19 pneumonia	Good COVID-19 outcome with pregnancy	[27]
Clinical profile, viral load, maternal-fetal outcomes of pregnancy with COVID-19: 4-week retrospective, tertiary care single-centre descriptive study	Single-center retrospective study	Most women with COVID-19 (78.9%) had a mild infection with favorable maternal–fetal outcomes	Better COVID-19 outcome with pregnancy	[21]
Asymptomatic SARS-CoV-2 infections in pregnant patients in an Italian city during the complete lockdown		Out of 325 asymptomatic pregnant women, six (1.8%) were positive; out of the six, none developed clinical symptoms	Good COVID-19 outcome with pregnancy	[23]
Clinical characteristics and intrauterine vertical transmission potential of COVID-19 infection in nine pregnant women: a retrospective review of medical records	Retrospective review of medical records	Clinical characteristics of COVID-19 pneumonia in pregnant women were like those reported for nonpregnant women	Better COVID-19 outcome with pregnancy	[28]
Routine screening for SARS CoV-2 in unselected pregnant women at delivery	Cross-sectional study	Nearly 50% of pregnant women were asymptomatic	Better COVID-19 outcome with pregnancy	[24]
Clinical findings and disease severity in hospitalized pregnant women with COVID-19	Prospective multicenter cohort study	Infection with COVID-19 caused moderate to severe respiratory illness among pregnant women	Worse COVID-19 outcome with pregnancy	[29]
Maternal and neonatal morbidity and mortality among pregnant women with and without COVID-19 infection: the INTERCOVID Multinational Cohort Study	Multinational cohort study	COVID-19 in pregnancy was associated with consistent and substantial increases in severe maternal morbidity and mortality and neonatal complications	Worse COVID-19 outcome with pregnancy	[30]

To begin with, a retrospective cohort study in Wuhan, China, reported an infection rate of SARS-CoV-2 among pregnant women (0.57%) to be comparable to that in the general population (0.50%) in Wuhan with no deaths among 11,078 confirmed COVID-19 cases that were used in the study [31]. Similarly, a study carried out in the Italian city of Genoa reported that six out of 325 screened asymptomatic pregnant women reporting for delivery tested positive for SARS-CoV-2. None of the SARS-CoV-2-positive women developed clinical symptoms, and no infection was reported in the newborns either [23]. Altogether, these findings suggest that pregnant women may not be at a higher risk of developing more severe COVID-19 complications compared with nonpregnant women.

Studies have reported severe COVID-19 outcomes among pregnant women, but these adverse outcomes appear to be associated with underlying conditions and not pregnancy *per se*. In support of this view, Allotey and colleagues found maternal risk factors associated with severe COVID-19 in pregnancy to include advanced age, high body mass index, chronic hypertension and pre-existing diabetes [10]. Pre-existing maternal comorbidity was found to be associated with admission to an intensive care unit and the need for invasive ventilation. Higher percentages of pregnancy obesity and gestational diabetes were also identified among pregnant women in eight US healthcare centers who were hospitalized for COVID-19 illness without an obstetric reason compared with those admitted for obstetric reasons [32]. Likewise, a national prospective cohort study among pregnant women in the UK reported that hospitalized pregnant women with symptomatic SARS-CoV-2 were more likely to be overweight or obese and to have a relevant medical comorbidity including asthma and hypertension compared with asymptomatic pregnant women [33]. Similarly, a case series from Italy reported obesity as the most important risk factor related to a severe form of COVID-19 in pregnant women [34].

Taken together, these reports seemingly demonstrate that pregnancy *per se* does not worsen COVID-19 outcome among pregnant women but rather confounding factors in pregnancy. Perhaps, correcting for these factors could provide a different outcome between COVID-19 pregnant and nonpregnant women, or the general population. It is quite likely that pregnant women without these underlying conditions are not disproportionately impacted by SARS-CoV-2.

## Physiological changes in pregnancy: beneficial or harmful in the context of COVID-19?

Maternal physiological adaptations during pregnancy are known to negatively impact the susceptibility of pregnant women to viral infections like SARS-CoV-2, as well as the clinical course of the infection [35]. The adaptations occur as a result of altered hormonal and metabolic cues that impact the size, morphology, function and responsiveness of tissues and organs, which manifest in alterations in the pulmonary, immune, cardiovascular and metabolic systems of the pregnant woman [36].

**Figure 1.**
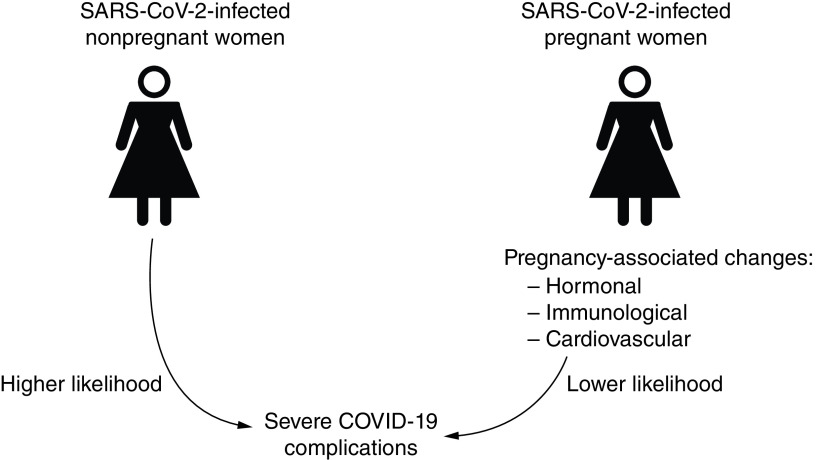
Illustration of the protective nature of pregnancy against severe COVID-19. Nonpregnant women have normal physiological functions hence rarely develop any adaptive physiological changes that may protect them against severe COVID-19. Pregnant women, however, tend to develop hormonal, immunological and cardiovascular changes that protect against adverse COVID-19 symptoms.

Physiological changes of the cardiothoracic region such as diaphragm elevation, lung expansion, increased oxygen consumption and hormone-induced edema of upper respiratory tract mucosa occur in pregnancy and result in pulmonary events such as reduced total lung capacity at term and poor clearance of pulmonary secretions [37,38]. Such pulmonary alterations may generally contribute to compromised functional ability and are closely linked to increased susceptibility and worsened morbidity of respiratory viral infections [18]. Thus, respiratory-related changes should be pertinent to severe cases of COVID-19 where pneumonia rapidly progresses from focal to diffuse bilateral consolidation of lung parenchyma [39], and thus is expected to readily predispose pregnant women to hypoxemic respiratory failure. However, most COVID-19 pregnant women have been shown to have less severe COVID-19 outcomes [21–23,26]. A summary of how pregnancy might be protective against severe COVID-19 is provided in Figure 1.

### Immunological adaptations of pregnancy & COVID-19

During pregnancy, there is a characteristic strong first-line response against viral pathogens that are mediated by the activation of natural killer cells and monocytes [40]. As pregnancy advances, there is a shift from inflammatory macrophages (M1) and natural killer cells to anti-inflammatory macrophages (M2), which promote Th2 response. The M2 secretes the anti-inflammatory cytokines TGF-β and IL-10, which remain elevated throughout the gestation period [41] and promote the healing of damaged tissue [42]. Therefore, the second line of defense is rather relatively defective due to the Th2 polarization phenomenon, a physiological shift from the dominant cell-mediated Th1 immunity to a dominant humoral Th2 immunity. The shift to the predominant Th2 immunity presents a high level of anti-inflammatory cytokines such as IL-4, IL-10, IL-13 and TGF-β and allows for immune tolerance of the developing semiallogeneic fetus while maintaining the ability to respond to pathogenic upset [43]. The function of the immune system-regulating T cells (the Treg lymphocytes) is also enhanced in pregnancy to maintain maternal immunological tolerance while further suppressing the proinflammatory Th1 and Th17 immune responses, and thereby promoting the Th1 to Th2 shift [44]. Additionally, the secretion of IFN-γ, a cytokine that is essential to both innate and adaptive immunity and functions in mediating antiviral immunity, has been shown to increase gradually as pregnancy progresses [45].

The immunologic adaptations during pregnancy could induce an increased susceptibility to intracellular pathogens including viruses, bacteria and parasites [46]. A study showed that pregnant women suffer greater viral infection-associated morbidity and mortality compared with nonpregnant women [17]. Although respiratory viral infections like SARS and MERS have had quite adverse outcomes among pregnant women, COVID-19 apparently presents a relatively favorable outcome. Case fatalities reported for COVID-19 pregnant women is approximately 0% compared with 18% and 25% for SARS and MERS, respectively, by the same period after disease onset [47–50]. A preferential activation of Th1 immunity at least 2 weeks after disease onset was observed among SARS patients [50], but COVID-19 patients presented both Th1 and Th2 immunities about the same period [48].

Taken together, it is likely that progressive respiratory failure, which is among the two most significant risk factors for fatality in SARS and MERS [47,50], is better regulated in SARS-CoV-2-infected pregnant women. The relatively predominant Th2 immunity over Th1 immune response in pregnant women may be a restraining factor against the cytokine storm and the fostering of immune protection against acute respiratory distress syndrome (ARDS) and multiorgan failure in COVID-19 [48].

### Hormonal changes in pregnancy & COVID-19

The leading driver of protective immunological adaptation in pregnancy is posited to be hormonal changes [45]. Alteration of hormonal patterns in pregnancy influences ventilatory changes and respiratory function [37]. Increased levels of progesterone, among other hormones, in pregnancy is reported to inhibit the extensive release of proinflammatory cytokines such as IL-6, IL-12, and TNF, which are among the primary triggers of adverse COVID-19 complications such as pneumonia, extensive pulmonary damage and the development of ARDS [51].

The role of progesterone in suppressing the induction of proinflammatory cytokine secretion in pregnancy and maintenance of the pregnancy by increasing Th2 cell cytokines production to predominate Th1 cell cytokine production within the decidua during early pregnancy is well documented [51,52]. Thus, the anti-inflammatory function of progesterone may be critical in preventing the adverse event of a cytokine storm, which is a key feature of severe COVID-19. Progesterone has also been reported to promote the healing of respiratory virus-induced lung damage [53].

Elevated estradiol levels are also reported to promote Th2 responses, whereas reduced levels of the hormone promote Th1 responses [54]. Future studies will need to verify the hormonal-related protective immunological response in gravidas with COVID-19. Progesterone and estrogen are also known to exert cardiovascular effects, and therefore alterations in levels of these hormones during pregnancy may be important in regulating cardiovascular comorbidities such as heart failure, coronary artery disease, hypertension and diabetes, which are high-risk factors for severe COVID-19 [55–57].

The importance of alterations in steroid hormones during pregnancy in women with COVID-19, vis-à-vis the underlying risk factors, will be worth studying particularly in comparison with nonpregnant women with similar cardiovascular and other underlying risk conditions.

### Cardiovascular changes in pregnancy & COVID-19

Several studies have associated COVID-19 with cardiovascular complications and reported pre-existing cardiovascular diseases to be among the highest risk factors of case fatalities [49,58,59]. However, only a few cases of COVID-19-related cardiomyopathy have been reported in pregnancy [60].

During pregnancy, alterations in the maternal cardiovascular system cause systemic vasodilation that further leads to increased gravid uterus and other organ perfusions, with reduced systemic vascular resistance accompanied by increase in cardiac output [61]. Elevated renal perfusion and glomerular filtration rates lead to upregulation of the renin–angiotensin–aldosterone system (RAAS) with associated plasma volume expansion [62,63], but blood pressure is not increased during normal pregnancy. Diastolic and systolic blood pressures have been shown to decrease by a mean of approximately 20% and 8%, respectively [64], which suggests possible cardioprotection in healthy pregnant women.

ACE2 and Ang 1–7 have been shown to be relatively increased in pregnancy [65]. ACE2/Ang 1–7 has antihypertensive and suppressive activities on myocardial proinflammatory cytokines TNFα and IL-6 [66,67]. The normal regulation against hypertension in pregnancy may potentially offer some protection against severe COVID-19 in pregnant women. ACE2/Ang 1–7-mediated cardioprotection has been shown via the association of reduced ACE2 with worsening of obesity-associated heart failure, adipose tissue inflammation and microvascular dysfunction [68]. ACE2 also converts angiotensin I to angiotensin 1–9 (Ang 1–9). However, the catalytic efficiency of ACE2 in using the Ang II to Ang 1–7 pathway is 400-fold higher than using the Ang I to Ang 1–9 pathway [69]. Therefore, a high level of ACE2, as occurs in pregnancy, will likely favor the Ang 1–7 pathway that promotes the maintenance of cardiovascular homeostasis thereby offering cardioprotective effects.

### Endothelial cell function & coagulability in COVID-19 pregnant women

Hypercoagulability has been suggested to be a critical consequence of severe SARS-CoV-2 [70], but pregnancy is already known to be a physiologically hypercoagulable condition [71]. In pregnancy, conditions such as preeclampsia contribute to endothelial cell dysfunction [72], which may occur as a result of high levels of circulating coagulation and fibrinolytic factors such as plasmin [73]. During normal pregnancy, there is an increase in the synthesis of the anti-inflammatory vasodilator nitric oxide (NO) [74] as a result of stimulation by progesterone. NO plays an important role in protecting against endothelial dysfunction and hypercoagulopathy via the relaxation of smooth muscles and modulation of vascular tone thereby regulating blood pressure [75]. This increased synthesis of NO during pregnancy may therefore offer some protection against endothelial dysfunction and hypercoagulopathy in COVID-19 pregnant women.

Since endothelial injury is part of the hallmarks of COVID-19, SARS-CoV-2 infection during pregnancy will be expected to aggravate complement activation and hypercoagulation, and portending an increased risk for severe endothelial, vascular and coagulopathic complications in COVID-19 pregnant women. However, COVID-19 pregnant women do not appear to have a significantly increased risk of thrombotic complications, probably due to some of the physiological adaptations in healthy pregnancy [76]. For instance, a study demonstrated that SARS-CoV-2 can directly infect endothelial cells via ACE2 and cause inflammation, thus altering the vascular homeostasis especially in severely sick persons [77]. ACE2 is internalized as a result, and thereby downregulated on endothelial cells after SARS-CoV-2 binds, promoting the pulmonary inflammatory and profibrotic processes via local Ang II activity [78]. In pregnancy, however, increased levels of ACE2 could convert the Ang II and avail high levels of Ang (1–7) which will act on the MAS receptor involved in inhibiting local prothrombotic endothelial phenotype in COVID-19 [79].

Normal regulation of coagulation inhibitors and blood clot-lytic enzymes of the endothelial cells are important for maintaining vessel wall integrity and balanced coagulation [80], and promote the development of a procoagulative endothelium, resulting from endothelitis and inflammatory cell infiltration, leading to initiation and propagation of ARDS in COVID-19 [81]. The RAAS and the kallikrein–kinin systems (KKS) work together to regulate the thromboresistance of endothelial cells [82–84], and reports suggest that vasodilation effects of relatively increased Ang 1–7 in pregnancy promote antithrombosis [85,86]. Reduced expression of ACE2 has been shown to cause an increase in vascular permeability via the activation of KKS [81,87], and thus an increased ACE2/Ang 1–7 activity in pregnancy may support vascular integrity, sustain impermeability and promote balanced coagulative endothelium via the MAS receptor and the KKS, thereby protecting COVID-19 pregnant women against ARDS.

The preceding discussion points to the protective effects of physiological changes in pregnant women against severe COVID-19. However, more detailed studies are warranted to decipher the exact mechanisms by which pregnant women are relatively better protected against severe COVID-19.

## Conclusion

Contrary to the general expectation that maternal physiological changes that occur in pregnancy could exacerbate the severity of COVID-19 in pregnant women, data on populations that develop severe disease are comparable between pregnant and nonpregnant women. Pregnant women tend to have immunological adaptations and cardiovascular and endothelial factors that appear to protect them against severe COVID-19. Further studies are needed to identify the exact mechanisms protecting pregnant women against severe COVID-19. The outcomes of such studies are likely to lead to more effective therapeutic and management options to complement the administration of COVID-19 vaccines in pregnant women.

## Future perspective

The COVID-19 pandemic has claimed many lives across the globe, especially among vulnerable groups such as the elderly and those with underlying comorbidities. Based on previous respiratory viral outbreaks, it was expected that pregnant women will be disproportionately affected by COVID-19. However, despite having conflicting reports, an appreciable number of studies have pointed to a less severe COVID-19 outcome among pregnant women. As suggested in this article, the physiological and immunological adaptations that occur during pregnancy are likely to be ameliorating the severity of COVID-19 in this cohort. We anticipate that this special report will stimulate studies that will be directed at understanding precisely how pregnancy is able to mitigate severe COVID-19 symptoms. With detailed research reports emerging on the mechanisms by which pregnancy downplays COVID-19 complications, we envisage identification of molecular players that protect pregnant women against severe COVID-19. Informed rationale design of effective treatment or management approaches that could help manage persons who have severe COVID-19 complications could be achieved, and such interventions are likely to be beneficial during COVID-19 and in the event of future respiratory-based pandemics.

**Executive summary**

**Background**

- Based on previous respiratory viral outbreaks, pregnant women were expected to be at higher risk of severe COVID-19 compared with the general population.

**Pregnancy-related risk factors for COVID-19**

- Pregnancy is associated with altered immune and respiratory functioning that predisposes pregnant women to severe COVID-19.

**Is pregnancy protective against severe COVID-19?**

- Appreciable data have shown that pregnant women without underlying comorbidities are not disproportionately affected by COVID-19 complications.

**Physiological changes in pregnancy: beneficial or harmful in the context of COVID-19?**

- Immunological and physiological adaptations during pregnancy appear to protect pregnant women from severe COVID-19 complications.
- A thorough understanding of the precise mechanism(s) by which pregnancy protects against severe COVID-19 complications is warranted.

## References

[1] CDC. People with Certain Medical Conditions (2020). www.cdc.gov/coronavirus/2019-ncov/need-extra-precautions/people-with-medical-conditions.html

[2] Englund JA, Chu HY. Respiratory virus infection during pregnancy: does it matter? J. Infect. Dis. 218(4), 512–515 (2018). 29741694 10.1093/infdis/jiy169 PMC7107415

[3] Mosby LG, Rasmussen SA, Jamieson DJ. 2009 pandemic influenza A (H1N1) in pregnancy: a systematic review of the literature. Am. J. Obstet. Gynecol. 205(1), 10–18 (2011). 21345415 10.1016/j.ajog.2010.12.033

[4] Siston AM, Rasmussen SA, Honein MA Pandemic 2009 influenza A(H1N1) virus illness among pregnant women in the United States. JAMA 303(15), 1517–1525 (2010). 20407061 10.1001/jama.2010.479 PMC5823273

[5] Cervantes-Gonzalez M, Launay O. Pandemic influenza A (H1N1) in pregnant women: impact of early diagnosis and antiviral treatment. Expert Rev. Anti. Infect. Ther. 8(9), 981–984 (2010). 20818941 10.1586/eri.10.83

[6] Shay M, MacKinnon AL, Metcalfe A Depressed mood and anxiety as risk factors for hypertensive disorders of pregnancy: a systematic review and meta-analysis. Psychol. Med. 50(13), 2128–2140 (2020). 32912348 10.1017/S0033291720003062

[7] Kotlar B, Gerson E, Petrillo S, Langer A, Tiemeier H. The impact of the COVID-19 pandemic on maternal and perinatal health: a scoping review. Reprod. Health 18(1), 10 (2021). 33461593 10.1186/s12978-021-01070-6 PMC7812564

[8] Ramsey PS, Ramin KD. Pneumonia in pregnancy. Obstet. Gynecol. Clin. North Am. 28(3), 553–569 (2001). 11512500 10.1016/s0889-8545(05)70217-5

[9] Rasmussen SA, Kissin DM, Yeung LF Preparing for influenza after 2009 H1N1: special considerations for pregnant women and newborns. Am. J. Obstet. Gynecol. 204(1 Suppl. 6), 13–20 (2011). 10.1016/j.ajog.2011.01.048 21333967

[10] Allotey J, Stallings E, Bonet M Clinical manifestations, risk factors, and maternal and perinatal outcomes of coronavirus disease 2019 in pregnancy: living systematic review and meta-analysis. BMJ 370, m3320 (2020). 32873575 10.1136/bmj.m3320 PMC7459193

[11] Berkowitz K, LaSala A. Risk factors associated with the increasing prevalence of pneumonia during pregnancy. Am. J. Obstet. Gynecol. 163(3), 981–985 (1990). 2403178 10.1016/0002-9378(90)91109-p

[12] Magro C, Mulvey JJ, Berlin D Complement associated microvascular injury and thrombosis in the pathogenesis of severe COVID-19 infection: a report of five cases. Transl. Res. 220, 1–13 (2020). 32299776 10.1016/j.trsl.2020.04.007 PMC7158248

[13] Jamieson DJ, Theiler RN, Rasmussen SA. Emerging infections and pregnancy. Emerg. Infect. Dis. 12(11), 1638–1643 (2006). 17283611 10.3201/eid1211.060152 PMC3372330

[14] Gardner MO, Doyle NM. Asthma in pregnancy. Obstet. Gynecol. Clin. North Am. 31(2), 385–413.vii (2004). 15200969 10.1016/j.ogc.2004.03.010

[15] Shah NM, Lai PF, Imami N, Johnson MR. Progesterone-related immune modulation of pregnancy and labor. Front. Endocrinol. (Lausanne) 10, 198 (2019). 30984115 10.3389/fendo.2019.00198 PMC6449726

[16] Shevyrev D, Tereshchenko V. Treg heterogeneity, function, and homeostasis. Front.Immunol. 10, 3100 (2020). 31993063 10.3389/fimmu.2019.03100 PMC6971100

[17] Racicot K, Mor G. Risks associated with viral infections during pregnancy. J. Clin. Invest. 127(5), 1591–1599 (2017). 28459427 10.1172/JCI87490 PMC5409792

[18] Li XC, Zhang J, Zhuo JL. The vasoprotective axes of the renin-angiotensin system: physiological relevance and therapeutic implications in cardiovascular, hypertensive and kidney diseases. Pharmacol. Res. 125, 21–38 (2017). 28619367 10.1016/j.phrs.2017.06.005 PMC5607101

[19] Hopkins E, Sharma S. Physiology, functional residual capacity. In: StatPearls. StatPearls Publishing, FL, USA (2022). www.ncbi.nlm.nih.gov/books/NBK500007/

[20] LoMauro A, Aliverti A. Respiratory physiology of pregnancy: physiology masterclass. Breathe 11(4), 297–301 (2015). 27066123 10.1183/20734735.008615 PMC4818213

[21] Bachani S, Arora R, Dabral A Clinical profile, viral load, maternal-fetal outcomes of pregnancy with COVID-19: 4-week retrospective, tertiary care single-centre descriptive study. J. Obstet. Gynaecol. Can. 43(4), 474–482 (2021). 33349556 10.1016/j.jogc.2020.09.021 PMC7591315

[22] Liu D, Li L, Wu X Pregnancy and perinatal outcomes of women with coronavirus disease (COVID-19) pneumonia: a preliminary analysis. Am. J. Roentgenol. 215(1), 127–132 (2020). 32186894 10.2214/AJR.20.23072

[23] Massarotti C, Adriano M, Cagnacci A Asymptomatic SARS-CoV-2 infections in pregnant patients in an Italian city during the complete lockdown. J. Med. Virol. 93(3), 1758–1760 (2021). 32841411 10.1002/jmv.26458 PMC7461157

[24] Díaz-Corvillón P, Mönckeberg M, Barros A Routine screening for SARS CoV-2 in unselected pregnant women at delivery. PLoS One 15(9), 1–13 (2020). 10.1371/journal.pone.0239887 PMC7524006 32991621

[25] Wang Y, Liang X, Wang H, Li L, Xiong G, Mi L. A considerable asymptomatic proportion and thromboembolism risk of pregnant women with COVID-19 infection in Wuhan, China. J. Perinat. Med. 49(2), 237–240 (2021). 33470960 10.1515/jpm-2020-0409

[26] Mattar CN, Kalimuddin S, Sadarangani SP Pregnancy outcomes in COVID-19: a prospective cohort study in Singapore. Ann. Acad. Med. Singap. 49(11), 857–869 (2020). 33381779

[27] Liu S, Dzakpasu S, Nelson C Pregnancy outcomes during the COVID-19 pandemic in Canada, March to August 2020. Journal of Obstetrics and Gynaecology Canada. 43(12), 1406–1415 (2021). 34332116 10.1016/j.jogc.2021.06.014

[28] Chen H, Guo J, Wang C Clinical characteristics and intrauterine vertical transmission potential of COVID-19 infection in nine pregnant women: a retrospective review of medical records. Lancet 395(10226), 809–815 (2020). 32151335 10.1016/S0140-6736(20)30360-3 PMC7159281

[29] Savasi VM, Parisi F, Patanè L Clinical findings and disease severity in hospitalized pregnant women with COVID-19. Obstetrics & Gynecology. 136(2), 252–258 (2020). 32433453 10.1097/AOG.0000000000003979

[30] Villar J, Ariff S, Gunier RB Maternal and neonatal morbidity and mortality among pregnant women with and without COVID-19 Infection: the INTERCOVID multinational cohort study. JAMA Pediatrics. 175(8), 817–826 (2021). 33885740 10.1001/jamapediatrics.2021.1050 PMC8063132

[31] Yang R, Mei H, Zheng T Pregnant women with COVID-19 and risk of adverse birth outcomes and maternal-fetal vertical transmission: a population-based cohort study in Wuhan, China. BMC Med. 18(1), 330 (2020). 33070775 10.1186/s12916-020-01798-1 PMC7568966

[32] Panagiotakopoulos L, Myers TR, Gee J SARS-CoV-2 infection among hospitalized pregnant women: reasons for admission and pregnancy characteristics – eight U.S. health care centers, March 1 – May 30, 2020. MMWR Morb. Mortal. Wkly. Rep. 69(38), 1355–1359 (2020). 32970660 10.15585/mmwr.mm6938e2 PMC7727498

[33] Vousden N, Bunch K, Morris E The incidence, characteristics and outcomes of pregnant women hospitalized with symptomatic and asymptomatic SARS-CoV-2 infection in the UK from March to September 2020: a national cohort study using the UK Obstetric Surveillance System (UKOSS). PLoS ONE 16(5), 1–19 (2021). 10.1371/journal.pone.0251123 PMC8099130 33951100

[34] Martino DD, Chiaffarino F, Patanè L Assessing risk factors for severe forms of COVID-19 in a pregnant population: a clinical series from Lombardy, Italy. Int. J. Gynaecol. Obstet. 152(2), 275–277 (2021). 33098568 10.1002/ijgo.13435 PMC9087617

[35] Maxwell C, McGeer A, Tai KFY, Sermer M. No. 225 – management guidelines for obstetric patients and neonates born to mothers with suspected or probable severe acute respiratory syndrome (SARS). J. Obstet. Gynaecol. Can. 39(8), e130–e137 (2017). 28729104 10.1016/j.jogc.2017.04.024 PMC7105038

[36] Napso T, Yong HEJ, Lopez-Tello J, Sferruzzi-Perri AN. The role of placental hormones in mediating maternal adaptations to support pregnancy and lactation. Front. Physiol. 9, 1091 (2018). 30174608 10.3389/fphys.2018.01091 PMC6108594

[37] LoMauro A, Aliverti A. Respiratory physiology of pregnancy: physiology masterclass. Breathe (Sheff). 11(4), 297–301 (2015). 27066123 10.1183/20734735.008615 PMC4818213

[38] Yan J, Guo J, Fan C Coronavirus disease 2019 in pregnant women: a report based on 116 cases. Am. J. Obstet. Gynecol. 223(1), 111.e1–111.e14 (2020). 32335053 10.1016/j.ajog.2020.04.014 PMC7177142

[39] Carotti M, Salaffi F, Sarzi-Puttini P Chest CT features of COVID-19 pneumonia: key points for radiologists. Radiol Med. 1–11 (2020). 32500509 10.1007/s11547-020-01237-4 PMC7270744

[40] Ghi T, di Pasquo E, Mekinian A, Calza L, Frusca T. Sars-CoV-2 in pregnancy: why is it better than expected? Eur. J. Obstet. Gynecol. Reprod. Biol. 252, 476–478 (2020). 32739606 10.1016/j.ejogrb.2020.07.025 PMC7378463

[41] Abdulamir AS, Hafidh RR. The possible immunological pathways for the variable immunopathogenesis of COVID-19 infections among healthy adults, elderly and children. Electron. J. Gen. Med. 17(4), 1–4 (2020).

[42] Robinson DP, Klein SL. Pregnancy and pregnancy-associated hormones alter immune responses and disease pathogenesis. Horm. Behav. 62(3), 263–271 (2012). 22406114 10.1016/j.yhbeh.2012.02.023 PMC3376705

[43] Aghaeepour N, Ganio EA, Mcilwain D An immune clock of human pregnancy. Sci. Transl. Med. 2(15), 1–12 (2017). 10.1126/sciimmunol.aan2946 PMC5701281 28864494

[44] Shevyrev D, Tereshchenko V. Treg heterogeneity, function, and homeostasis. Front. Immunol. 10(Jan.), 1–13 (2020). 10.3389/fimmu.2019.03100 PMC6971100 31993063

[45] Abu-Raya B, Michalski C, Sadarangani M, Lavoie PM. Maternal immunological adaptation during normal pregnancy. Front. Immunol. 11, 575197 (2020). 33133091 10.3389/fimmu.2020.575197 PMC7579415

[46] Mor G, Cardenas I. The immune system in pregnancy: a unique complexity. Am. J. Reprod. Immunol. 63(6), 425–433 (2010). 20367629 10.1111/j.1600-0897.2010.00836.x PMC3025805

[47] Assiri A, Abedi GR, Al Masri M, Bin Saeed A, Gerber SI, Watson JT. Middle East respiratory syndrome coronavirus infection during pregnancy: a report of 5 cases from Saudi Arabia: table 1. Clin. Infect. Dis. 63(7), 951–953 (2016). 27358348 10.1093/cid/ciw412 PMC5812010

[48] Huang C, Wang Y, Li X Clinical features of patients infected with 2019 novel coronavirus in Wuhan, China. Lancet 395(10223), 497–506 (2020). 31986264 10.1016/S0140-6736(20)30183-5 PMC7159299

[49] Long B, Brady WJ, Koyfman A, Gottlieb M. Cardiovascular complications in COVID-19. Am. J. Emerg. Med. 38(7), 1504–1507 (2020). 32317203 10.1016/j.ajem.2020.04.048 PMC7165109

[50] Wong SF, Chow KM, Leung TN Pregnancy and perinatal outcomes of women with severe acute respiratory syndrome. Am. J. Obstet. Gynecol. 191(1), 292–297 (2004). 15295381 10.1016/j.ajog.2003.11.019 PMC7137614

[51] Shah NM, Lai PF, Imami N, Johnson MR. Progesterone-related immune modulation of pregnancy and labor. Front. Endocrinol. 10(198), 1–19 (2019). 10.3389/fendo.2019.00198 PMC6449726 30984115

[52] Piccinni MP. T cells in normal pregnancy and recurrent pregnancy loss. Reprod. Biomed. Online 13(6), 840–844 (2006). 17169207 10.1016/s1472-6483(10)61033-4

[53] Hall OJ, Klein SL. Progesterone-based compounds affect immune responses and susceptibility to infections at diverse mucosal sites. Mucosal Immunol. 10(5), 1097–1107 (2017). 28401937 10.1038/mi.2017.35

[54] Kourtis AP, Read JS, Jamieson DJ. Pregnancy and Infection. N. Engl. J. Med. 370(23), 2211–2218 (2014). 24897084 10.1056/NEJMra1213566 PMC4459512

[55] Zhu L, She ZG, Cheng X Association of blood glucose control and outcomes in patients with COVID-19 and pre-existing type 2 diabetes. Cell Metab. 31(6), 1068–1077.e3 (2020). 32369736 10.1016/j.cmet.2020.04.021 PMC7252168

[56] Guan WJ, Liang WH, He JX, Zhong NS. Cardiovascular comorbidity and its impact on patients with COVID-19. Eur. Respir. J. 55(6), 2001227 (2020). 32341104 10.1183/13993003.01227-2020 PMC7236831

[57] Li B, Yang J, Zhao F Prevalence and impact of cardiovascular metabolic diseases on COVID-19 in China. Clin. Res. Cardiol. 109(5), 531–538 (2020). 32161990 10.1007/s00392-020-01626-9 PMC7087935

[58] Lang JP, Wang X, Moura FA, Siddiqi HK, Morrow DA, Bohula EA. A current review of COVID-19 for the cardiovascular specialist. Am. Heart J. 226, 29–44 (2020). 32497913 10.1016/j.ahj.2020.04.025 PMC7252118

[59] Iqubal A, Iqubal MK, Hoda F, Najmi AK, Haque SE. COVID-19 and cardiovascular complications: an update from the underlying mechanism to consequences and possible clinical intervention. Exp. Rev. Anti Infect. Ther. 19(9), 1083–1092 (2021). 10.1080/14787210.2021.1893692 PMC7938651 33618607

[60] Nejadrahim R, Khademolhosseini S, Kavandi H, Hajizadeh R. Severe acute respiratory syndrome coronavirus-2- or pregnancy-related cardiomyopathy, a differential to be considered in the current pandemic: a case report. J. Med. Case Rep. 15(1), 143 (2021). 33741059 10.1186/s13256-021-02751-3 PMC7978166

[61] Soma-Pillay P, Nelson-Piercy C, Tolppanen H, Mebazaa A. Physiological changes in pregnancy. CVJA 27(2), 89–94 (2016). 27213856 10.5830/CVJA-2016-021 PMC4928162

[62] Tkachenko O, Shchekochikhin D, Schrier RW. Hormones and hemodynamics in pregnancy. Int. J. Endocrinol. Metab. 12(2), e14098 (2014). 24803942 10.5812/ijem.14098 PMC4005978

[63] Ames MK, Atkins CE, Pitt B. The renin-angiotensin-aldosterone system and its suppression. J. Vet. Intern. Med. 33(2), 363–382 (2019). 30806496 10.1111/jvim.15454 PMC6430926

[64] Hermida RC, Ayala DE, Mojón A Blood pressure patterns in normal pregnancy, gestational hypertension, and preeclampsia. Hypertension 36(2), 149–158 (2000). 10948070 10.1161/01.hyp.36.2.149

[65] Tamanna S, Clifton VL, Rae K, van Helden DF, Lumbers ER, Pringle KG. Angiotensin converting enzyme 2 (ACE2) in pregnancy: preeclampsia and small for gestational age. Front. Physiol. 11, 590787 (2020). 33101066 10.3389/fphys.2020.590787 PMC7554608

[66] Yamazato M, Yamazato Y, Sun C, Diez-Freire C, Raizada MK. Overexpression of angiotensin-converting enzyme 2 in the rostral ventrolateral medulla causes long-term decrease in blood pressure in the spontaneously hypertensive rats. Hypertension 49(4), 926–931 (2007). 17325232 10.1161/01.HYP.0000259942.38108.20

[67] Kong L, Sattentau QJ. Antigenicity and immunogenicity in HIV-1 antibody-based vaccine design. J. AIDS Clin. Res. 2012(Suppl. 8), 003 (2012). 10.4172/2155-6113 PMC3515071 23227445

[68] Patel R, Gupta A, Chauhan S, Bansod DW. Effects of sanitation practices on adverse pregnancy outcomes in India: a conducive finding from recent Indian demographic health survey. BMC Pregnancy Childbirth 19(1), 378 (2019). 31651276 10.1186/s12884-019-2528-8 PMC6813085

[69] Vickers C, Hales P, Kaushik V Hydrolysis of biological peptides by human angiotensin-converting enzyme-related carboxypeptidase. J. Biol. Chem. 277(17), 14838–14843 (2002). 11815627 10.1074/jbc.M200581200

[70] Kichloo A, Dettloff K, Aljadah M COVID-19 and hypercoagulability: a review. Clin. Appl. Thromb. Hemost. 26, 1076029620962853 (2020). 33074732 10.1177/1076029620962853 PMC7592310

[71] Soma-Pillay P, Catherine NP, Tolppanen H, Mebazaa A, Tolppanen H, Mebazaa A. Physiological changes in pregnancy. Cardiovasc. J. Afr. 27(2), 89–94 (2016). 27213856 10.5830/CVJA-2016-021 PMC4928162

[72] Burton GJ, Redman CW, Roberts JM, Moffett A. Pre-eclampsia: pathophysiology and clinical implications. BMJ 366, 1–15 (2019). 10.1136/bmj.l2381 31307997

[73] Ji HL, Zhao R, Matalon S, Matthay MA. Elevated plasmin(ogen) as a common risk factor for COVID-19 susceptibility. Physiol. Rev. 100(3), 1065–1075 (2020). 32216698 10.1152/physrev.00013.2020 PMC7191627

[74] You Y, Tan W, Guo Y Progesterone promotes endothelial nitric oxide synthase expression through enhancing nuclear progesterone receptor-SP-1 formation. Am. J. Physiol. Heart Circ. Physiol. 319(2), H341–H348 (2020). 32618512 10.1152/ajpheart.00206.2020

[75] Ahmad A, Dempsey SK, Daneva Z Role of nitric oxide in the cardiovascular and renal systems. Int. J. Mol. Sci. 19(9), 2605 (2018). 10.3390/ijms19092605 PMC6164974 30177600

[76] Wastnedge EAN, Reynolds RM, van Boeckel SR Pregnancy and COVID-19. Physiol. Rev. 101(1), 303–318 (2021). 32969772 10.1152/physrev.00024.2020 PMC7686875

[77] Monteil V, Kwon H, Prado P Inhibition of SARS-CoV-2 infections in engineered human tissues using clinical-grade soluble human ACE2. Cell 181(4), 905–913.e7 (2020). 32333836 10.1016/j.cell.2020.04.004 PMC7181998

[78] Verdecchia P, Cavallini C, Spanevello A, Angeli F. The pivotal link between ACE2 deficiency and SARS-CoV-2 infection. Eur. J. Intern Med. 76, 14–20 (2020). 32336612 10.1016/j.ejim.2020.04.037 PMC7167588

[79] Perico L, Benigni A, Casiraghi F, Ng LFP, Renia L, Remuzzi G. Immunity, endothelial injury and complement-induced coagulopathy in COVID-19. Nat. Rev. Nephrol. 17(1), 46–64 (2021). 33077917 10.1038/s41581-020-00357-4 PMC7570423

[80] Van Hinsbergh VWM. Endothelium – role in regulation of coagulation and inflammation. Semin. Immunopathol. 34(1), 93–106 (2012). 21845431 10.1007/s00281-011-0285-5 PMC3233666

[81] Teuwen LA, Geldhof V, Pasut A, Carmeliet P. COVID-19: the vasculature unleashed. Nat. Rev. Immunol. 20(7), 389–391 (2020). 32439870 10.1038/s41577-020-0343-0 PMC7240244

[82] Stavrou EX, Fang C, Merkulova A Reduced thrombosis in Klkb1-/- mice is mediated by increased Mas receptor, prostacyclin, Sirt1, and KLF4 and decreased tissue factor. Blood 125(4), 710–719 (2015). 25339356 10.1182/blood-2014-01-550285 PMC4304115

[83] Schmaier AH. The contact activation and kallikrein/kinin systems: pathophysiologic and physiologic activities. J. Thromb. Haemost. 14(1), 28–39 (2016). 26565070 10.1111/jth.13194

[84] Schmaier AH. A novel antithrombotic mechanism mediated by the receptors of the kallikrein/kinin and renin-angiotensin systems. Front. Med. (Lausanne) 3, 61 (2016). 27965959 10.3389/fmed.2016.00061 PMC5124569

[85] Santos RAS, Sampaio WO, Alzamora AC The ACE2/angiotensin-(1-7)/MAS axis of the renin-angiotensin system: focus on angiotensin-(1-7). Physiol. Rev. 98(1), 505–553 (2018). 29351514 10.1152/physrev.00023.2016 PMC7203574

[86] Li M, Chen L, Zhang J, Xiong C, Li X. The SARS-CoV-2 receptor ACE2 expression of maternal-fetal interface and fetal organs by single-cell transcriptome study. PLoS ONE 15(4), e0230295 (2020). 32298273 10.1371/journal.pone.0230295 PMC7161957

[87] Garvin MR, Alvarez C, Miller JI A mechanistic model and therapeutic interventions for COVID-19 involving a RAS-mediated bradykinin storm. eLife 9, e59177 (2020). 32633718 10.7554/eLife.59177 PMC7410499

